# Cost-effectiveness and budget impact of venetoclax in combination with rituximab in relapsed/refractory chronic lymphocytic leukemia in Switzerland

**DOI:** 10.1007/s10198-021-01398-7

**Published:** 2021-11-10

**Authors:** Michaela Barbier, Nicholas Durno, Craig Bennison, Mathias Örtli, Christian Knapp, Matthias Schwenkglenks

**Affiliations:** 1 Institute of Pharmaceutical Medicine (ECPM), University of Basel, Klingelbergstrasse 61, 4056 Basel, Switzerland; 2 OPEN Health, Oxford, UK; 3 AbbVie AG, Alte Steinhauserstrasse 14, 6330 Cham, Switzerland

**Keywords:** Rituximab, Venetoclax, Chronic lymphocytic leukemia, Cost-effectiveness, Partitioned survival model, EVPI, 119

## Abstract

**Introduction:**

Venetoclax in combination with rituximab (VEN + R) demonstrated prolonged overall survival (OS) and progression-free survival (PFS) for patients with relapsed/refractory (R/R) chronic lymphocytic leukemia (CLL) in comparison to standard chemoimmunotherapy [bendamustine + rituximab (BR)]. We conducted a cost-effectiveness and budget impact analysis comparing VEN + R versus six comparators from the Swiss healthcare payer perspective.

**Methods:**

A three-state partitioned survival model, developed in accordance with NICE and ISPOR decision modelling guidelines, was adapted to Switzerland. Model inputs were informed by the MURANO trial (survival data, patient characteristics), publicly available Swiss sources (drug prices, inpatient and outpatient costs), Swiss National Institute of Cancer Epidemiology and Registration data (incidence and prevalence values), and Swiss medical expert feedback. We used published (dis-)utility values and adverse event probabilities.

**Results:**

Over a lifetime, VEN + R resulted in an expected gain of 2.60 quality-adjusted life years (QALYs) per patient and incremental costs of Swiss Francs (CHF) 147,851 compared to BR, leading to an incremental cost-effectiveness ratio of CHF 56,881/QALY gained. Other treatment strategies (for example ibrutinib versus VEN + R) resulted in higher costs and lower QALYs. Results were not different for subgroups of patients with/without deletion of chromosome 17p/tumour protein 53 mutation. In scenario analysis, changes in post-progression treatment costs demonstrated a high impact on results. We estimated an expected value of perfect information of CHF 3,318/patient. A moderate VEN + R uptake was estimated to save CHF 12.3 million during 5 years.

**Conclusions:**

Using a threshold of CHF 100,000 per QALY, VEN + R was projected to be cost-effective vs BR.

**Supplementary Information:**

The online version contains supplementary material available at 10.1007/s10198-021-01398-7.

## Introduction

Chronic lymphocytic leukemia (CLL) is a malignancy of the B-cell lymphocytes in the blood, bone marrow and secondary lymphoid tissue [1]. It is the most common type of leukemia in Western countries with an incidence of 4.2/100,000 population/year [2]. CLL is most prominent in the older population, often having clinically relevant coexisting conditions [3, 4]. Relapsed or refractory (R/R) CLL remains incurable, despite advances in treatment [5, 6]. Current treatment options for R/R CLL disease include chemotherapy (e.g. fludarabine, bendamustine) in combination with rituximab, the oral B-cell receptor inhibitors idelalisib in combination with rituximab, ibrutinib (in combination with rituximab and bendamustine or alone) [3], or the oral B-cell lymphoma 2-inhibitor venetoclax (alone or in combination with rituximab).

In the current study, we evaluated the cost-effectiveness and budget impact of 2-year treatment with venetoclax and rituximab (VEN + R) in R/R CLL versus six comparators (fludarabine + cyclophosphamide + rituximab (FCR) and bendamustine + rituximab (BR) for a maximum of six cycles; ibrutinib, ibrutinib + BR, idelalisib + R, and venetoclax monotherapy (VEN) until disease progression) from a Swiss statutory health insurance perspective.

The only randomised controlled trial of VEN + R is the phase 3 international multicentre MURANO trial {NCT02005471 [5]}. The trial compared VEN + R with BR for R/R CLL patients. To estimate the relative efficacy of VEN + R with the other comparators identified as relevant for this study, a matching adjusted indirect treatment comparison (MAIC) was used. It utilised data from trials in which the comparator treatments were administered (4-year ibrutinib outcomes from RESONATE [6, 7], study 116 for idelalisib + R [8, 9], HELIOS for ibrutinib + BR [10], 10-year data from Badoux et al. [11] for FCR, and pooled patient-level data of a phase 1 and phase 2 study [12, 13] for VEN monotherapy). Except for VEN monotherapy, the trials were identified from a systematic literature review.

## Methods

### Model structure and overall approach

The cost-effectiveness analysis was based on the Swiss adaptation of a three-state partitioned survival model (with health states of progression-free survival (PFS), post-progression survival (PPS) and death) provided by AbbVie, and developed in Microsoft Excel by OPEN Health in accordance with National Institute of Health and Clinical Care Excellence (NICE) [14, 15] and International Society for Pharmacoeconomics and Outcomes Research (ISPOR) decision modelling guidelines [16]. Currently, the model is only fully described in the NICE committee papers for the Single Technology Appraisal of VEN [17, 18]. Patients were distributed between the three states using parameterized survival curves estimated from clinical trial results. Survival curves were estimated through (1) joint modelling of overall survival (OS) and PFS using individual patient data (for VEN + R and BR) and (2) from digitalisation and MAIC of published survival curves (for the remaining comparators). More details are provided in the section “Survival modelling”.

For the adaptation to Switzerland, we examined if Swiss specific parameter values existed. Resource use values were verified with three Swiss medical experts in haematology (public hospital doctor, outpatient practitioner, private hospital doctor). We sourced costs from publicly available Swiss sources [19–22], and applied published utility values [17]. In the base-case analysis, we used a time horizon of 30 years to represent the lifetime of patients, a cycle length of 28 days with half cycle correction applied and an annual discount rate of 3% for costs and quality-adjusted life years (QALYs). All analyses were carried out from the Swiss statutory health insurance perspective.

For the budget impact analysis, we adapted the 5-year budget impact model developed by OPEN Health to reflect the situation in Switzerland. We estimated the annual expected number of patients treated with R/R CLL in Switzerland and assigned different market shares to the subpopulations with and without deletion of chromosome 17p/tumour protein 53 mutation del(17p)/TP53, which were based on AbbVie internal projections for the years 2021–2025.

### Population

In general, the modelled population was consistent with the MURANO trial eligibility criteria and patient characteristics [5]; i.e. patients had relapsed/refractory CLL following prior first-line (1L) treatment, an average baseline age of 64.2 years, and a body surface area of 1.92 m^2^ (the latter adapted to Switzerland [23, 24], DuBois Formula). Three quarters (73.8%) of patients were male, 27.0% had del(17p)/TP53 (AbbVie data on file for deletion percentage).

### Intervention

The intervention treatment was VEN + R, which comprised of a titration phase and a post-titration phase as per the MURANO protocol [5]. The titration phase involved VEN being administered orally and daily for 5 weeks, with the dose gradually escalated on a weekly basis from 20 mg/day in the first week to 400 mg/day in week 5. Following this, in the post-titration phase patients received VEN orally and daily with a 400 mg/day dose, for a maximum of 2 years. Hence, VEN was discontinued at 2 years or when patients progressed, whichever occurred earlier. In the post-titration phase, patients were also administered rituximab intravenously (IV) for six 28-day cycles, as a 375 mg/m^2^ dose on day 1 of the first cycle, and a 500 mg/m^2^ dose on day 1 of cycles 2–6.

### Comparators

The six comparator strategies were ibrutinib, idelalisib + R, FCR, BR, ibrutinib + BR, and VEN monotherapy [5–13, 25]. Bendamustine, rituximab, fludarabine, and cyclophosphamide are fixed dose treatments, administered for a maximum of six cycles, whereas the remaining treatments are administered until disease progression (VEN monotherapy, ibrutinib, idelalisib). Supplementary Table S1 provides more details on drug dosing schedules.

VEN monotherapy is only reimbursed in Switzerland for the subgroup of patients with del(17p)/TP53 after Bruton tyrosine kinase inhibitor (BTKi) failure [21]. Also, ibrutinib + BR is not reimbursed for patients with R/R CLL. Only for completeness, we generated overall and subgroup analysis results including the VEN monotherapy and the ibrutinib + BR comparators.

### Main outcomes

For the cost-effectiveness analysis (CEA), we calculated costs, QALYs, net monetary benefit (NMB) and incremental cost-effectiveness ratios (ICERs) in the form of incremental cost per QALY gained, after eliminating dominated treatment strategies from the comparison of the multiple treatment options. We hence applied a standard rational choice approach to compare multiple treatment options {as e.g. outlined in Glick et al. [26]}. The resulting ICERs were compared with a willingness-to-pay (WTP) threshold of Swiss Francs (CHF) 100,000/QALY, which is sometimes tentatively considered in analyses for Switzerland [27–29]. In an additional analysis, we defined an “average comparator” as a weighted average of the mean costs and QALYs of all six comparators considered in the analysis. We compared the costs and QALYs of VEN + R with this “average comparator”. The weights used were current market share estimates for these treatments (Supplementary Table S2). Market shares were based on AbbVie internal estimations for the end of the year 2020 which were also used for the starting year of the 5 year budget impact analysis (BIA). For the BIA, we estimated the difference in costs (undiscounted in CHF) between a world with and without VEN + R treatment.

### Model inputs

#### Survival modelling

To specify the distribution of patients across health states in each cycle of the partitioned survival model, we fitted extrapolations to the VEN + R and BR survival data from the MURANO trial. The follow-up period over which we could obtain data from the trial was 4 years (second data cut—July 2018), meaning that sizeable extrapolations were necessary to extend outcome estimates over the lifetime of patients. To estimate clinically plausible long-term survival, a joint modelling approach was pursued. It assumed proportionality between overall and progression-free survival endpoints and the treatments included in the MURANO trial. This method, along with stringent testing of the assumptions, led to long-term estimates that we considered clinically plausible [17]. A parameterization with the Weibull distribution was selected based on the Akaike information criteria, Bayesian information criteria and clinical plausibility [17]. The remaining comparator survival curves (except for BR) were constructed using OS and PFS hazard ratios (HRs) from anchored and unanchored MAIC comparisons, and coupling these with the estimated VEN + R parametric survival curves, while assuming the proportional hazards (PH) assumption to hold {[17], Supplementary Table S3}. In scenario analyses, we relaxed the PH assumption and applied individually estimated comparator survival curves, based on digitalised curves generated from published Kaplan–Meier data [7–11, 30] and parameterized with a Weibull distribution. The data source for VEN monotherapy consisted of selectively matched patients from the M12-175 and M13-982 trials [12, 13]. No patients from the M14-032 trial [25] were included, as this trial exclusively enrolled B-cell receptor inhibitor failure patients. In two different scenario analyses, unadjusted and adjusted individually estimated OS and PFS curves were used. For the latter, we reweighted the curves with the results of the MAIC, so that the individually estimated survival curves for the comparator reflected what would have occurred if the comparator treatment had been administered to the MURANO population.

#### Utilities

Swiss utility values for R/R CLL patients were not available, and individual patient EQ-5D-3L data collected in the MURANO trial were considered as heavily skewed towards the upper value of 1 and thus not reflective of the real world [17]. For this reason, the VEN + R NICE submission utility values (pre-progression 0.748, post-progression 0.600) were applied [17]. These values were obtained from previous NICE technology appraisals of VEN {TA487 [31]} and idelalisib + R {TA359 [32]}. In addition, utilities were adjusted by a multiplicative age-related deterioration factor for both pre- and post-progression health states (Supplementary Tables S4–S5).

#### Adverse events

We considered the utility impact of treatment emergent adverse events (AEs) of grades 3–4 which occurred in  ≥ 5% of patients in relevant trials {VEN + R and BR [33], ibrutinib [6], idelalisib + R [8, 9], FCR [11], VEN studies M12-175/ M13-982/M14-032 [12, 13, 25], ibrutinib + BR [10]}, as outlined in Supplementary Table S6. The impact of each AE on QALYs was incorporated by assigning a utility decrement and a duration estimate to each AE. The estimates were based on previous NICE technology appraisals and the literature {Supplementary Table S7, [17]}.

#### Medical resource use

Full details on drug dosing and routes of administration are provided in Supplementary Table S1. Since VEN can cause rapid death of CLL cells and tumour reduction, there is potential for tumour lysis syndrome (TLS). Therefore, all VEN patients were assumed to receive a TLS prophylaxis regimen before the initial dose of VEN which was adjusted according to their TLS risk level. We assumed 18.06% of patients to be at a lower TLS risk (based on criteria applied to MURANO patient-level data: node diameter  < 5 cm and absolute lymphocyte count < 25 × 10^9^)], and the remaining 79.94% of VEN patients to be at a higher TLS risk (out of which 32.20% had a creatinine clearance ≥ 80 millilitre per minute (mL/min), and 49.74% had a lower creatinine clearance). Supplementary Table S8 provides more details about resource use for TLS prophylaxis and monitoring (per creatine clearance, per lower and higher risk group).

With regard to routine care and monitoring, after discussions with the clinical expert group, we assumed this would comprise full blood counts, lactate dehydrogenase (LDH) tests, and haematologist visits (Table 1).

**Table 1:** Summary of cost data used in the cost-effectiveness model

Drug costs	Pack cost	Source
Venetoclax	CHF 99.10	Venclyxto^®^ (14 × 10 mg) [21]
	CHF 223.15	Venclyxto^®^ (7 × 50 mg) [21]
	CHF 429.95	Venclyxto^®^ (7 × 100 mg) [21]
	CHF 843.50	Venclyxto^®^ (14 × 100 mg) [21]
	CHF 6,153.75	Venclyxto^®^ (112 × 100 mg) [21]
Rituximab	CHF 1,225.70	Rixathon^®^, Truxima^®^ (500 mg) [21]
Ibrutinib	CHF 5,927.65	Imbruvica^®^ (28 × 240 mg) [21]
Idelalisib	CHF 4,052.00	Zydelig^®^ (60 × 150 mg) [21]
Bendamustine	CHF 212.45	Bendamustin Accord^®^ and Sandoz^®^ (100 mg) [21]
Fludarabine	CHF 171.85	Fludarabin Teva^®^, Accord^®^ (50 mg) [21]
Cyclophosphamide	CHF 20.00	Endoxan^®^ (500 mg) [21]

Drug administration costs	Costs per visit	Description and source
Rituximab (first infusion)	CHF 629.55	On day 1 of first cycle [19]
Rituximab (infusions 2–6)	CHF 246.35	On day 1 of cycles 2 to 6 [19]
Bendamustine	CHF 203.77	On days 1 and 2 of each cycle [19]
Fludarabine	CHF 203.77	On days 1, 2, and 3 of each cycle [19]
Cyclophosphamide	CHF 203.77	On days 1, 2, and 3 of each cycle [19]

TLS prophylaxis and monitoring costs	Costs during first cycle	Source
Lower risk patients	CHF 1,699	[19–22, 34]
Higher risk patients	CHF 9,941	

Routine care and monitoring costs	Unit cost	Resource use {cost sources [19, 20]}
Full blood count	CHF 37.80	Annually pre-progression: 4, post-progression: 8
Lactate dehydrogenase	CHF 2.50	Annually pre-progression: 4, post-progression: 0
Haematologist visit	CHF 172.82	Annually pre-progression: 4 visits, post-progression: 4 visits

Adverse event costs	Unit cost	Comment and source
Anaemia	CHF 1,205	[19–21, 35]
Febrile neutropenia	CHF 5,741	[22], Assumption 100% of cases are inpatient (medical expert feedback)
Infusion-related reaction	CHF 383	TARMED, outpatient only
Pneumonia	CHF 2,079	[22], Assumption 33% of cases are inpatient (medical expert feedback)
Thrombocytopenia	CHF 1,832	[19–21, 35]

Terminal care costs	Per patient	Source
Terminal care costs	CHF 17,340.16	[28]

*CHF* Swiss Francs, *IV* intravenous, *mg* milligram, *SL* Specialty list

#### Costs

We included costs (in CHF) of active treatment, treatment administration, routine care, and monitoring (stratified by PFS and PPS state), TLS prophylaxis and monitoring, AEs, post-progression treatment, and terminal care. Drug wastage costs were incorporated in the base case; thereby assuming that no vial sharing in Switzerland occurs. Drug prices, inpatient and outpatient unit costs were obtained from publicly available Swiss sources [19–22] as detailed in Table 1.

In the base case cost-effectiveness model, only costs for one line of treatment were included [corresponding to the second-line (2L) of treatment given the R/R status of the population studied]. We undertook a scenario analysis in which a possible set of further line treatments in Switzerland were included for all strategies except for VEN monotherapy {based on clinical expert opinion, Swiss specialty list reimbursement restriction for VEN monotherapy and Onkopedia CLL guideline [21, 36]}. VEN monotherapy reimbursement is restricted to R/R CLL patients with a prior failure of a B-cell receptor inhibitor and del(17p)/TP53 mutation (Supplementary Table S9). It hence represents a last line treatment option. We included third-line treatment costs on a per-cycle basis, as shown in Supplementary Table S10.

#### Budget impact analysis (BIA) inputs

In 2019, there were 8,603,900 residents living permanently in Switzerland. There was a growth in the Swiss permanent resident population of 0.7% between 2018 and 2019. CLL incidence (5.11 per 100,000 population per year) and prevalence values (51.69/100,000 population) were provided by the Swiss National Institute of Cancer Epidemiology and Registration (NICER) [37], with further details shown in Supplementary Table S11. Current and projected market shares were based on AbbVie internal estimates and are outlined in Supplementary Table S2. Market shares for VEN monotherapy for patients without del(17p)/TP53 and for ibrutinib + BR were set to zero, as the drugs are not reimbursed in Switzerland for this indication as previously mentioned.

### Uncertainty analyses

To investigate parameter and structural uncertainty in the CEA, we performed deterministic and probabilistic analyses (PA), as well as several scenario analyses. We varied all individual parameters with the exception of drug costs. For the PA, we compared all treatment strategies among each other and assigned beta distributions to rates and utilities, gamma distributions to costs and resource use estimates, and normal distributions to the logged HRs. When there were no standard error estimates available for a given parameter, the standard error was assumed to be 10% of the mean estimate unless stated otherwise. 10,000 simulations were performed.

For the deterministic analyses, we compared VEN + R with each comparator. Available 95% confidence intervals (CIs) were generally used to define the maximum and minimum boundaries. Where CIs were not available, we applied the 2.5% and 97.5% quantiles of the distributions assigned for the PA. Results were presented as tornado plots, displaying the six most influential parameters.

Finally, in scenario analyses, in which VEN + R was compared to each comparator strategy, we varied discount rates, time horizon, utility values, adverse event rates, rituximab mode of administration, survival distributions for OS/PFS extrapolation, used individually estimated survival curves instead of joint survival curves, used observed MURANO time on treatment (ToT) (instead of MURANO protocol ToT), included further line treatments, and varied the costs for TLS, routine care and terminal care. Scenario analysis details are presented in Supplementary Table S12. We also analysed subgroups of patients with and without del(17p)/TP53, as the prognosis of the disease is assumed to be worse when the mutation is present [2, 38]. While immunoglobulin heavy chain variable (IGHV) mutational status is a prognostic marker (unmutated IGHV is associated with a shorter remission time following initial treatment), we do not consider patients with an unmutated IGHV status as the sole risk factor to qualify for high or very-high risk categories. Therefore, no further subgroup analysis related to IGHV mutational status was performed. Finally, we used the expected value of perfect information (EVPI) approach to assess whether further clinical research to reduce parameter uncertainty may be worthwhile.

For the BIA, we undertook scenario analyses to address uncertainty (Supplementary Table S13). For instance, we investigated the influence on budget impact from using a different set of market shares for all treatment strategies, with a high market share of VEN + R. We also varied routine costs of care between -20% and + 20%, TLS prophylaxis costs between − 50% and + 50%, and we halved, doubled and removed adverse event probabilities. Next to the combined incident and prevalent population used in the base case, we restricted in further scenario analyses the population to prevalent and incident patients only.

## Results

### Cost-effectiveness analysis

Over a lifetime and after discounting, the VEN + R strategy relative to the BR strategy was projected to generate incremental costs of CHF 147,851 and incremental QALYs of 2.60 (3.73 QALYs undiscounted, 5.51 LYs undiscounted), resulting in an ICER of CHF 56,881 per QALY gained (Table 2). We found that VEN + R was a dominant strategy when compared to VEN monotherapy, ibrutinib, or ibrutinib + BR. Ibrutinib led to higher lifetime costs but lower benefits measured in QALYs when compared to VEN + R and VEN monotherapy.

**Table 2:** Base-case ICER results

Treatment	Costs (CHF)	QALYs	NMB^1^ (CHF)	Comment	Δ Cost (CHF)	Δ QALYs	ICERs (CHF per QALY gained) of non-dominated strategies
BR	42,004	3.981	356,116				Reference
FCR	43,637	2.914	247,760	Dominated by BR			
Idelalisib + R	114,677	2.479	133,226	Dominated by BR, FCR			
VEN + R	189,855	6.581	468,198		147,851	2.599	56,881
VEN	275,857	4.541	178,237	Dominated by VEN + R			
Ibrutinib	375,534	4.450	69,480	Dominated by VEN + R, VEN			
Ibrutinib + BR	504,245	5.303	26,023	Dominated by VEN + R			

*B* Bendamustine, *C* Cyclophosphamide; *CHF* Swiss Francs, *F* Fludarabine, *ICER* Incremental cost-effectiveness ratio, *NMB* Net monetary benefit, *QALY* Quality-adjusted life year, *R* Rituximab, *VEN* Venetoclax

^1^Assuming a WTP threshold of CHF 100,000 / QALY

For most treatment strategies, the most substantial cost components were the costs of the drugs themselves. Specifically, 95% of the total costs for ibrutinib, 95% for ibrutinib + BR, 89% for VEN, 83% for VEN + R, 81% for idelalisib + R, 37% for BR, and 34% for FCR, were drug acquisition costs (Supplementary Table S14).

Results were stable in subgroups of patients with/without del(17p)/TP53 (Supplementary Tables S15 and S16). When we compared VEN + R versus the weighted “average comparator” reflecting current Swiss market shares, VEN + R was dominant. Lifetime (30 year) mean costs per patient were CHF 305,421 for the average comparator versus CHF 189,855 for VEN + R. Lifetime mean QALYs were substantially lower for the average comparator (4.07) than for VEN + R (6.58).

In deterministic sensitivity analysis, changes in utility parameter values, VEN + R/BR joint model OS/PFS hazard rates, and OS and PFS HRs, had the most substantial impact on ICER and NMB outcomes (Supplementary Fig. S1 for VEN + R vs BR). ICERs for the VEN + R strategy versus the comparator strategies remained below CHF 100,000 per QALY gained even when pessimistic assumptions regarding model input parameter values were implemented.

From the scenario analyses, we observed that the inclusion of post-progression treatment costs and changes to the time horizon were most impactful on the ICER results (Supplementary Tables S17, S18 and S19). Changes to the discount rates for costs and QALYs were moderately influential. Shorter time horizons led to less favourable ICERs for the VEN + R treatment strategy. For the comparison of VEN + R versus BR, time horizons of 10 years or less resulted in ICERs above CHF 100,000 per QALY gained. This was because VEN + R accumulated fewer incremental QALYs over the shorter time horizons, while the majority of VEN + R costs were accrued within the first 2 years. For the scenario in which the Swiss specific set of further line treatments was incorporated (Supplementary Table S17), we found BR to be dominated by VEN, VEN to produce an ICER of CHF 54,975 per QALY gained relative to FCR, and VEN + R to produce an ICER of CHF 77,286 per QALY gained relative to VEN.

**Fig. 1:**
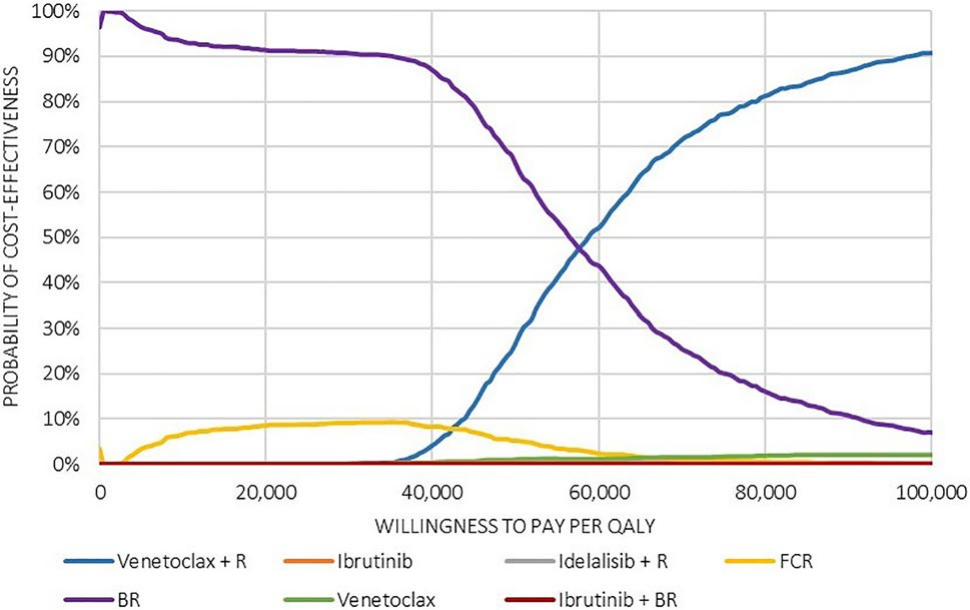
Cost-effectiveness acceptability curve. *B* Bendamustine, *C* Cyclophosphamide, *CHF* Swiss Francs, *QALY* Quality-adjusted life year, *R* Rituximab, *VEN* Venetoclax

In the PSA, we varied model parameters simultaneously. For the FCR, BR and VEN + R treatment strategies, changes in incremental QALYs were more pronounced than changes in incremental costs, which is mainly due to the fact that these treatments are stopped after a fixed duration, providing less room for cost variation. At a WTP threshold of CHF 100,000 per QALY, VEN + R was by far the most likely strategy to be cost-effective, based on 10,000 simulated runs (Fig. 1). With perfect information, we estimated a net benefit of CHF 470,070, leading to an EVPI (opportunity loss) of CHF 3,318 per patient.

### Budget impact analysis

At current Swiss prices, the availability of VEN + R was projected to reduce Swiss health care spending by CHF 12.3 million over 5 years, starting at the beginning of the year 2021 (Table 3). Highest savings were estimated for drug costs (CHF 14.3 million) and terminal care costs (CHF 2.9 million). In contrast, costs were expected to rise for TLS prophylaxis (CHF 3.7 million). The budget impact was estimated to be positive (implying net costs) during the first 2 years, but expected to be negative in each of the years 3–5. The main reason for this is that VEN if combined with rituximab has a maximum treatment period of 2 years. On a per-patient level, anticipated cost savings were CHF 5,912 per patient over 5 years (Supplementary Table S20).

**Table 3:** Budget impact by cost category over 5 years

Cost category	World with VEN + R (CHF)	World without VEN + R (CHF)	Budget impact (CHF)
Total costs	264.3 million	276.6 million	− 12.3 million
Active treatment	244,569,292	258,834,452	− 14,265,161
Treatment administration	1,954,195	1,574,947	379,248
Wastage	1,001,684	720,567	281,117
PFS routine care and monitoring costs	3,844,079	3,256,357	587,723
PPS routine care and monitoring costs	944,153	1,199,955	− 255,801
Terminal care costs	7,036,962	9,894,279	− 2,857,317
Treatment specific monitoring (TLS)	4,378,148	695,763	3,682,385
Adverse events	564,041	425,704	138,337

*CHF* Swiss Franc, *PFS* Progression-free survival, PPS Post-progression survival, *R* Rituximab, *TLS* Tumour lysis syndrome, *VEN* Venetoclax

In scenario analyses for the BIA, we observed that increasing or decreasing the price of venetoclax by 20% led to significant changes in estimated costs in years 1 and 2, and smaller changes in years 3, 4, and 5, where a smaller group of patients would be on treatment (Supplementary Table S21). Also, restricting the treated population to the incident population, led to a budget impact (cost increase) by CHF 5.2 million over 5 years as opposed to cost savings for the prevalent and incident population (when comparing a world with VEN + R to a world without). This was due to the advantage of limited VEN + R treatment duration being less pronounced, given that treatment initiation was roughly equally spread over the selected 5-year time horizon for patients in this scenario. Variation in the routine costs of care or adverse event rates had a negligible effect on BIA results, whereas changes in TLS costs and the size of the treated R/R population resulted in small to moderate changes in the incremental budget.

## Discussion

We found that a 2-year fixed duration treatment of VEN + R in R/R CLL patients may represent a cost-effective use of resources in the Swiss statutory health insurance system, and may be associated with costs savings of CHF 12.3 million over a 5-year period (starting from the beginning of 2021). A previous analysis for the United Kingdom (UK) undertaken for the regulator NICE [17], estimated that VEN + R treatment in the UK was a dominant strategy when compared with ibrutinib containing regimens, and estimated ICERs for VEN + R of Great Britain Pound (GBP) 16,865 relative to idelalisib + R, GBP 36,603 relative to FCR, and GBP 44,778 per QALY gained relative to BR. Internal AbbVie/OPEN Health estimations predicted that VEN + R will generate a cumulative cost impact if made available in the UK, of GBP 28.5 million over 5 years. In contrast, we estimated in this study that overall cost savings can be expected in Switzerland when switching from a world without to a world with VEN + R. This difference may in part be attributable to differences in market shares of R/R CLL treatments between the UK and Switzerland, as well as predicted market uptake of VEN + R. We expect the market uptake for VEN + R to be much higher in Switzerland (ranging from 25 to 60%) than in the UK (ranging from 11.83 to 19.67%).

In general, our cost-effectiveness analysis found VEN + R to be cost-effective relative to BR and less costly but more effective than ibrutinib. A hypothetical time limitation of ibrutinib treatment would most likely reduce its costs, but costs would likely still be higher and benefits still lower than for treatment with VEN + R. Where novel agents are not accessible, BR may remain a valuable treatment option with good overall tolerability, especially for low-risk R/R CLL patients that experience a late relapse upon 1L chemoimmunotherapy. It is also worth mentioning that in Switzerland, access to 2L ibrutinib is limited to patients who relapse early on 1L treatment.

There are currently no peer reviewed articles which present cost-effectiveness results for VEN + R in R/R CLL. Abstracts and a poster currently exist for adaptations of the model forming the basis of this analysis, for the USA [18, 39] and Argentina [40]. The adaptation for the USA from a US payer perspective also showed VEN + R to be cost-effective versus BR [18, 39], and a dominant strategy relative to other comparators. The adaptation for Argentina, compared VEN + R to ibrutinib only, using a social security payer perspective and a discount rate for costs and QALYs of 5% annually [40]. In this analysis, VEN + R was also a dominant strategy relative to ibrutinib.

Strengths of the analysis are the use of a three-health state partitioned survival model constructed according to NICE and ISPOR guidelines and implemented for Switzerland, as well as the use of patient-level data from the MURANO trial for the VEN + R and BR strategies. In addition, we performed a value of information analysis to assess the usefulness of conducting further studies of VEN + R and its comparators for R/R CLL to resolve cost-effectiveness uncertainty. Our results indicate that further research for this purpose may not be warranted.

Another strength of our analysis was the use of Swiss data to the extent possible [20–22] and consultation with Swiss medical experts to verify or obtain resource use values that were appropriate for Switzerland. CLL incidence and prevalence values were provided by an official source in Switzerland (NICER) [37].

There are several limitations to our analysis related to uncertainties about the model structure and input parameter values. The follow-up period from the MURANO trial was 4 years, meaning that sizeable extrapolations were necessary to estimate costs and QALYs over the lifetime of patients. A further limitation is the joint modelling of the PFS and OS curve parameters for VEN + R and BR for the base-case analysis and its assumption of proportional hazards. The assumption seems reasonable for the observed data but is unknown for extrapolations. On the other hand, the joint modelling approach was considered optimal for this study as it leveraged the more mature PFS data to inform the OS extrapolations, and gave the most clinically plausible OS estimates at 20 years for VEN + R versus other considered survival models [17]. In the scenario analysis in which individually estimated curves for all comparator strategies including BR were used, only small to moderate changes in the ICER results were generated.

Another limitation is that survival curves for other comparators were estimated based on a mostly disconnected evidence network, including many different trials with populations that may not be totally comparable. For comparisons without an anchor, matching adjusted ITC had to be implemented to adjust for observable trial differences, but such an approach does not adjust for unobservable characteristics. Another limitation is that OS and PFS results obtained from potentially highly selected trial populations may not be completely generalisable to the real world setting.

A further model limitation is related to post-progression treatments. The base-case analysis covered one line of treatment only (which corresponded to 2L treatment of patients with R/R CLL). A Swiss specific set of further line (3L) drug and administration costs was included in a scenario analysis based on feedback from one medical expert with experience gained in private and public hospitals. It naturally follows that the results of this scenario analysis are only valid for the assumed 3L treatment set. Guidelines had also been consulted but did not recommend a specific post-progression treatment scheme after a relapse in 2L in Switzerland. There was consensus among the three medical experts we consulted that 3L treatment is patient specific and dependent on the observed efficacy and tolerability of 1L and 2L treatment, genetic aberrations (del(17p)/TP53mut, complex karyotype), comorbidities and patient performance status. In this scenario analysis, VEN + R was still a cost-effective strategy if assuming a WTP threshold of CHF 100,000 per QALY gained. OS and PFS survival estimates used in the base case may have been influenced by further line treatments in case of longer-term follow-up in clinical trials. Scenario analysis results may potentially be even more realistic than the base-case results.

A cost component we had no information for were outpatient costs of terminal care; to mimic this, inpatient terminal care costs were increased in a scenario analysis resulting in a negligible impact on the ICER.

A further, potential limitation is the use of non-Swiss data sources when Swiss information was not available. This was the case for survival curve parameters, adverse event probabilities, utility values, and TLS risk distribution. Utility values were not taken from the MURANO trial but sourced from external studies, and were the same utility values that were used for the NICE submission in the UK for VEN monotherapy. Although utility values elicited from Swiss patients may be different to utility values elicited in other settings, we do not expect this factor to influence our results substantially.

## Conclusion

VEN + R appears to be a cost-effective treatment option for R/R CLL patients in Switzerland if a cost-effectiveness threshold of CHF 100,000 per QALY gained is assumed. VEN + R resulted in an ICER of CHF 56,881 per QALY gained vs BR. Other treatment options were dominated (i.e. more costly and less effective). Similar results were seen in subgroups defined by del(17p)/TP53 mutation status. In the budget impact analysis, VEN + R was estimated to result in savings of CHF 12.3 million during the first 5 years following introduction for R/R CLL patients in the Swiss statutory health insurance system.

## Supplementary Information

Below is the link to the electronic supplementary material.

Supplementary file1 (PDF 654 KB)
