# Supplementary material for: Cost-effectiveness and budget impact of venetoclax in combination with rituximab in relapsed/refractory chronic lymphocytic leukemia in Switzerland
Source: Eur J Health Econ. 2021 Nov 10;23(5):837–46. doi: 10.1007/s10198-021-01398-7 (PMC9170651; doi:10.1007/s10198-021-01398-7)
Supplement: Supplementary file 1 — Supplementary file1 (PDF 654 KB) [file 10198_2021_1398_MOESM1_ESM.pdf]

Cost-effectiveness and budget impact of venetoclax in combination with rituximab in relapsed/refractory chronic lymphocytic leukemia in Switzerland

The European Journal of Health Economics

Michaela Barbier, Nicholas Durno, Craig Bennison, Mathias Oertli, Christian Knapp, Matthias Schwenkglenks

Corresponding author:

Dr. Michaela Carla Barbier, Institute of Pharmaceutical Medicine, University of Basel,

Klingelbergstrasse 61, CH-4056 Basel, Switzerland. E-mail: michaela.barbier@unibas.ch

Electronic Supplementary Materials

Supplementary Tables and Figures

Table S1 Treatment regimens

Regimen	Drug	Admin	Dosing schedule
**VEN+R**	Venetoclax	Oral	Daily dose, 20 mg week 1, 50 mg week 2, 100mg week 3, 200 mg week 4, 400 mg week 5 and 400 mg from week 6 until disease progression or for 2-year maximum treatment duration.
	Rituximab	IV	375 mg/m² D1 C1, 500 mg/m² D1 C2-C6 for a total of 6 doses.
**Ibrutinib**	Ibrutinib	Oral	Daily dose of 420 mg until disease progression
**Idelalisib + R**	Idelalisib	Oral	Daily dose of 300 mg until disease progression
	Rituximab	IV	375 mg/m² D1 C1, 500 mg/m² D1 C2-C6 for a total of 6 doses.
**FCR**	Fludarabine	IV	25 mg/m² D1-D3 C1-C6 for a total of 18 doses
	Cyclophosphamide	IV	250 mg/m² D1-D3 C1-C6 for a total of 18 doses
	Rituximab	IV	375 mg/m² D1 C1, 500 mg/m² D1 C2-C6 for a total of 6 doses
**BR**	Bendamustine	IV	90 mg/m² D1-D2, C1-C6 for a total of 12 doses
	Rituximab	IV	375 mg/m² D1 C1, 500 mg/m² D1 C2-C6 for a total of 6 doses
**VEN**	Venetoclax	Oral	Daily dose, 20 mg week 1, 50mg week 2, 100mg week 3, 200mg week 4, 400 mg week 5 and beyond
**Ibrutinib + BR**	Ibrutinib	Oral	Daily dose of 420 mg until disease progression
	Bendamustine	IV	90 mg/m² D2–D3 C1, and D1-D2 C2–C6 for a total of 12 doses
	Rituximab	IV	375 mg/m² D1 C1, 500 mg/m² D1 C2-C6 for a total of 6 doses

Abbreviations: *B* Bendamustine, *C* Cyclophosphamide, *Cx* cycle, *Dx* day x, *F* Fludarabine, *IV* Intravenous, *R* Rituximab, *VEN* Venetoclax

Table S2 Swiss market shares for current and projected treatment landscapes (estimated by AbbVie)

	Non-del(17p)/TP53					Del(17p)/TP53				
**World without VEN+R**	**2021**	**2022**	**2023**	**2024**	**2025**	**2021**	**2022**	**2023**	**2024**	**2025**
**VEN+R**	0.0%	0.0%	0.0%	0.0%	0.0%	0.0%	0.0%	0.0%	0.0%	0.0%
**Ibrutinib**	57.0%	57.0%	57.0%	57.0%	57.0%	62.0%	59.0%	59.0%	59.0%	59.0%
**Idelalisib+R**	22.0%	22.0%	22.0%	22.0%	22.0%	20.0%	16.0%	16.0%	16.0%	16.0%
**FCR**	4.0%	4.0%	4.0%	4.0%	4.0%	0.0%	0.0%	0.0%	0.0%	0.0%
**BR**	17.0%	17.0%	17.0%	17.0%	17.0%	0.0%	0.0%	0.0%	0.0%	0.0%
**VEN**	0.0%	0.0%	0.0%	0.0%	0.0%	18.0%	25.0%	25.0%	25.0%	25.0%
**Ibrutinib+BR**	0.0%	0.0%	0.0%	0.0%	0.0%	0.0%	0.0%	0.0%	0.0%	0.0%
**World with VEN+R**	**2021**	**2022**	**2023**	**2024**	**2025**	**2021**	**2022**	**2023**	**2024**	**2025**
**VEN+R**	28.0%	38.0%	47.0%	55.0%	60.0%	25.0%	31.0%	36.0%	41.0%	46.0%
**Ibrutinib**	43.0%	38.0%	35.0%	33.0%	30.5%	55.0%	53.5%	52.0%	50.0%	46.0%
**Idelalisib+R**	8.0%	7.0%	6.0%	4.0%	4.0%	8.0%	7.5%	7.0%	6.0%	6.0%
**FCR**	4.0%	3.0%	2.0%	1.0%	0.5%	0.0%	0.0%	0.0%	0.0%	00%
**BR**	17.0%	14.0%	10.0%	7.0%	5.0%	0.0%	0.0%	0.0%	0.0%	0.0%
**VEN**	0.0%	0.0%	0.0%	0.0%	0.0%	12.0%	8.0%	5.0%	3.0%	2.0%
**Ibrutinib+BR**	0.0%	0.0%	0.0%	0.0%	0.0%	0.0%	0.0%	0.0%	0.0%	0.0%

Abbreviations: *B* Bendamustine, *C* Cyclophosphamide, *CHF* Swiss Francs, *R* Rituximab, *VEN* Venetoclax

Table S3 OS and PFS hazard ratios

	PFS			OS		
**Treatment**	**Hazard ratio vs comparator**	**95% LCI**	**95% UCI**	**Hazard ratio vs comparator**	**95% LCI**	**95% UCI**
Ibrutinib	0.80	0.53	1.20	0.45	0.25	0.80
Idelalisib+R	0.17	0.09	0.34	0.19	0.07	0.53
FCR	0.32	0.21	0.50	0.24	0.12	0.47
BR	0.19	0.14	0.27	0.51	0.30	0.86
VEN – del(17p)[1]	0.44	0.22	0.91	0.51	0.18	1.42
Ibrutinib + BR	1.19	0.68	2.10	0.59	0.22	1.57

[1]Due to the small sample size of Non-Del(17p) patients, this comparison is associated with a large degree of uncertainty and is therefore not suitable for use within the economic model. For the Non-del(17p) analysis it is assumed the relative efficacy would be the same as the Del(17p) population.
Included studies in the MAIC: [1-10].
Abbreviations: *B* Bendamustine, *C* Cyclophosphamide, *CI* Confidence interval, *del(17p)* Deletion of short arm of chromosome 17p, *F* Fludarabine, *LCL* Lower confidence interval, *MAIC* matched adjusted indirect treatment comparison, *R* Rituximab, *UCL* Upper confidence interval *VEN* Venetoclax

Source: NICE [11]

Table S4 Health state utility values

Health state	Utility	Source
Pre-progression	0.748	EQ-5D data study 116 [10, 12]
Post-progression	0.600	Dretzke et al. 2010 [13]

Source: NICE [11, 12, 14]

Table S5 Age related utility adjustment

Age bracket	N	Mean	95% CI	Adjustment from baseline
**60-≤65**	2739	0.8072	(0.793,0.821)	1
**66-≤70**	2993	0.8041	(0.790,0.817)	0.996
**71-≤75**	2501	0.779	(0.766,0.791)	0.965
**76-≤80**	1895	0.7533	(0.739,0.767)	0.933
**81-≤85**	1199	0.6985	(0.677,0.719)	0.865
**86+**	655	0.6497	(0.624,0.675)	0.805
**Total**	11,982			

Abbreviations: *CI* confidence interval, *N* Sample size

Source: NICE [11]

Table S6 Adverse event probabilities (grades 3-4)

Adverse event	VEN+R	Ibrutinib	Idelalisib+R	FCR	BR	VEN	Ibrutinib + BR
ALT/AST elevation	1.55%		5.45%		1.60%		
Anaemia	10.82%	4.62%	5.45%	25.59%	13.83%	13.78%	3.48%
Anaemia (Autoimmune haemolytic)	2.58%				1.60%	5.33%	
Febrile neutropenia	3.61%				9.57%	4.44%	11.85%
Infusion related reaction	2.06%				5.32%		1.39%
Neutropenia	58.76%	16.41%	33.64%	41.94%	39.89%	39.11%	53.66%
Pneumonia	6.19%	6.67%			7.98%	6.67%	7.32%
Thrombocytopenia	6.19%	5.64%	10.00%	24.89%	10.11%	13.33%	14.98%
N	194	195	110	284	188	330	287
Source	MURANO [1]	RESONATE [6]	Study 116 [10]	FCR study [7]	MURANO [1]	VEN trials M12-175/ M13-982/ M14-032 [2-4]	HELIOS [8]

Abbreviations: *ALT* Alanine Transaminase, *AST* Aspartate Transaminase, *B* Bendamustine, *C* Cyclophosphamide, *F* Fludarabine, *N* Sample size, *R* Rituximab, *VEN* Venetoclax

Source: NICE [11]

Table S7 Averse event disutility, duration and QALY decrements

Adverse event	Disutility Value	Duration (months)	QALY decrement
ALT/AST elevation	0.050	0.690	0.003
Anaemia	0.090	0.763	0.006
Anaemia (autoimmune haemolytic)	0.090	0.763	0.006
Febrile neutropenia	0.195	0.763	0.012
Infusion related reaction	0.200	0.115	0.002
Neutropenia	0.163	0.496	0.007
Pneumonia	0.195	0.598	0.010
Thrombocytopenia	0.108	0.763	0.007

Abbreviations: *ALT* Alanine Transaminase, *AST* Aspartate Transaminase

Source: NICE [11]

Table S8 Resource use for TLS prophylaxis and monitoring (per patient)

Week	1	2	3	4	5	Total
**Lower Risk**						
Renal function panel[2]	1	1	1	1	1	5
Uric acid test	3	3	3	3	3	15
Allopurinol (mg)	3,000	2,100	2,100	2,100	2,100	11,400
Rasburicase (mg)	0	0	0	0	0	0
Rasburicase infusion	0	0	0	0	0	0
1.5L Sodium chloride 0.9%	0	0	0	0	0	0
CT Scan	1	0	0	0	0	1
Inpatient day	0	0	0	0	0	0
Office visit (outpatient)	1	1	1	1	1	5
**Higher Risk**						
Renal function panel*	3[1]	3	3	3	3	15
Uric acid test	3[1]	3	3	3	3	15
Allopurinol (mg)	4,200[1]	2,100	2,100	2,100	2,100	12,600
Rasburicase (mg)	0.38[1,3]	0	0	0	0	0.38
Rasburicase infusion	0.02[1]	0	0	0	0	0.02
1.5L Sodium chloride 0.9% per week	1[1]	1	1	1	1	5
CT Scan	1	0	0	0	0	1
Inpatient day	1	0	0	0	0	1
Office visit (outpatient)	0	1	1	1	1	4

[1]Inpatient resource use: costs covered by the inpatient DRG reimbursement.

[2] Blood Urea Nitrogen, Creatinine, Phosphate, Albumin, Bicarbonate, Laktat-Dehydrogenase (LDH), Chloride, Calcium, Natrium/Sodium, Kalium/Potassium

[3]30.64 mg * 2.03% * 49.74% / 81.94%

Abbreviations: *CT* Computer Tomography, *L* Litre, *TLS* Tumour Lysis Syndrome

Table S9 Post-progression treatments in scenario analysis

2L treatment		Post-progression treatment	Source
VEN+R	→	Idelalisib+R	Medical expert feedback, [15, 16]
BR	→	ibrutinib monotherapy (100%)	MURANO
Idelalisib+R	→	Idelalisib (double dose, i.e. 600 mg per day) (36.4% of the patients based on study 116)	[12]
Ibrutinib	→	VEN+R (80%), VEN unlimited (10%), idelalisib+R (10%)	Medical expert feedback, [15, 16]
Ibrutinib+BR	→	VEN unlimited (50%), idelalisib+R (50%)	Medical expert feedback, [15, 16]
FCR	→	Ibrutinib (50%), VEN+R (50%)	Medical expert feedback, [15, 16]
VEN monotherapy	→	No further line assumed	Medical expert feedback, [15, 16]

Abbreviations: *B* Bendamustine, *C* Cyclophosphamide, *HDMP* High dose methylprednisolone, *R* Rituximab, *VEN* Venetoclax

Table S10 Post-progression treatment costs in scenario analysis

Second-line treatment	Per cycle post-progression (Third-line) treatment costs
VEN+R	CHF 3,968
Ibrutinib	CHF 6,328
Idelalisib+R	CHF 2,750
FCR	CHF 6,296
BR	CHF 5,928
VEN monotherapy	CHF 0
Ibrutinib+BR	CHF 5 249

Abbreviations: *B* Bendamustine, *BSA* Body surface area, *C* Cyclophosphamide, *CHF* Swiss Francs, *R* Rituximab, *VEN* Venetoclax

Table S11 Budget impact model inputs and data sources

Parameter	Value	Reference
Swiss population (end of 2019)	8,603,900	SFSO [17]
Annual population growth	0.7%	SFSO [18]
CLL prevalence rate (per 100,000)	51.69/100,000 (0.052%)	NICER (cancer types C830 and C911). Point prevalence with reference date 31.12.2016
CLL age-adjusted incidence rate (per 100,000)	5.11/100,000 (0.005%)	NICER (diagnosis period 2012-2016)
Percentage of treated R/R CLL	21.6%	[19, 20]
Percentage with del(17p)/TP53 mutation	26.96%	MURANO trial

Abbreviations: *CLL* Chronic lymphocytic leukaemia, *NICER* National Institute of Cancer Epidemiology and Registration, *R/R* Relapsed/refractory

Table S12 Scenario analyses (cost-effectiveness model)

Scenario	Description
Discount rates	The discount rates associated with costs and outcomes was varied between 0% and 6%
Time horizon	The time horizon was set to 1, 2, 5, 10, 15, 20 and 25 years
VEN+R survival models	Generalised gamma, gamma, log-logistic, log-normal survival models were used as an alternative to the Weibull model for the joint model by endpoint and treatment
Comparator survival curves	In this scenario, the comparator survival curves were estimated through individual rather than through joint curve estimation. In the first scenario, individual curves were estimated without adjustment. In the second scenario, individual curves were estimated with adjustment, by using the results of the MAIC to reweight the characteristics of the VEN+R baseline sample to approximately match with the comparator baseline characteristics.
VEN TLS prophylaxis costs	The TLS prophylaxis costs were halved, doubled and removed
Routine Costs of Care	The routine costs of pre- and post-progression care were halved, doubled and removed. Secondly, resource use frequencies from the ibrutinib NICE submission were used
Terminal care costs	Terminal care costs were increased by 5%, 10%,15% and 20%
Time on Treatment (ToT)	In the base-case analysis, we assumed treatment would occur for the durations which are stipulated in the treatment protocols of BR and VEN+R. However, in real clinical practice, patients may choose to discontinue treatment before receiving the full course, or clinicians may not strictly adhere to the treatment protocols for these regimens. These aspects were reflected in a scenario analysis, where we instead use real data from the MURANO trial from which ToT Kaplan Meier curves were constructed, in order to make estimations of the proportion of patients remaining on VEN+R and BR treatment over time
Rituximab administration method	Only 'faster' infusion for R treatment were assumed
Adverse events	Adverse event rates were halved, doubled and removed
Costs of next line treatment	Further line treatment for overall 59.4% of the patients was included (AbbVie data on file). The set of 3L treatments was outlined in supplementary Table S9
Utilities	First, alternative pre-and post-progression utility values were tested. Second, the difference between pre-and post-progression utility values was varied between 0.1 and 0.5. Third, adverse event disutilities were doubled and removed. Finally, the difference between VEN+R and BR EQ-5D-3L utility values, based on the MURANO trial, was used to adjust the PFS utility value

Abbreviations: *B Bendamustine*, *OS* Overall survival, *PFS* Progression-free Survival, *R* Rituximab, *TLS* Tumour lysis syndrome, *VEN* Venetoclax

Table S13 Budget impact scenario analyses

Scenario	Description
VEN price	The per mg price of VEN was varied between +20% and -20% (+5%, +10%, +15%, +20%, -5%, -10%, -15%, -20%)
Routine costs of care	The routine costs of care were varied between +20% and -20% (+5%, +10%, +15%, +20%, -5%, -10%,- 15%, -20%).
Adverse events	Adverse event probabilities were halved, doubled and removed
TLS prophylaxis costs	The costs of TLS prophylaxis were varied between +50% and -50%
BIA population	The R/R population was increased and decreased by 10%.<br>In separate scenarios, the populations were restricted to “prevalent only” and “incident only”.

Abbreviations: *BIA* Budget impact analysis, *mg* Milligram, *R/R* Relapsed/Refractory, *TLS* Tumour lysis syndrome, *VEN* Venetoclax

Table S14 Base case lifetime cost results per treatment strategy (discounted, in CHF)

Treatment	Active treatment	Treatment Admin	Wastage	PFS health state costs	PPS health state costs	Terminal care costs	Monitoring (TLS)	Adverse events	Total
**BR**	15,491	3,741	1,711	1,476	4,547	14,083	24	931	42,004
**FCR**	14,856	8,153	1,243	2,153	1,727	15,150	24	331	43,637
**Idelalisib + R**	92,782	1,742	1,146	1,385	2,108	15,415	24	75	114,677
**VEN + R**	156,786	1,832	1,178	4,532	4,678	11,872	8,467	511	189,855
**VEN monotherapy**	246,551	0	0	2,649	3,802	13,762	8,456	636	275,857
**Ibrutinib**	355,561	0	0	3,920	1,777	14,053	24	199	375,534
**Ibrutinib + BR**	477,124	4,245	1,866	5,066	1,607	13,419	24	893	504,245

Abbreviations: *B* Bendamustine, *C* Cyclophosphamide; *CHF* Swiss Francs, *F* Fludarabine, *PFS* Progression-free survival, *PPS* Post-progression survival, *R* Rituximab, *VEN* Venetoclax

Table S15 Base case ICER lifetime results (non-dominated strategies), del(17p)/TP53 subgroup

Treatment	Costs	QALYs	LYs*	Δ Cost	Δ QALY	Δ LYs*	Pairwise ICER
BR	CHF 41,461	3.585	6.571	CHF 144,824	2.442	5.112	**CHF 59,315**
VEN + R	CHF 186,285	6.026	11.684				

Abbreviations: *B* Bendamustine, *CHF* Swiss Francs, *F* Fludarabine, *ICER* Incremental cost-effectiveness ratio, *LY* Life years, *QALY* Quality-adjusted life year, *R* Rituximab, *VEN* Venetoclax
*undiscounted

Table S16 Base case ICER lifetime results (non-dominated strategies), non-del(17p)/TP53 subgroup

Treatment	Costs	QALYs	LYs*	Δ Cost	Δ QALY	Δ LYs*	Pairwise ICER
BR	CHF 42,189	4.136	7.736	CHF 148,793	2.652	5.634	**CHF 56,114**
VEN + R	CHF 190,982	6.788	13.370				

Abbreviations: *B* Bendamustine, *CHF* Swiss Francs, *F* Fludarabine, *ICER* Incremental cost-effectiveness ratio, *LY* Life years, *QALY* Quality-adjusted life year, *R* Rituximab, *VEN* Venetoclax
*undiscounted

Table S17 Scenario results including further line treatment

Treatment	Costs (in CHF)	QALYs	NMB[1] (in CHF)	Comment	ICER[2]
FCR	186,414	2.914	104,983		Reference
Idelalisib + R	190,807	2.479	57,096	Dominated by FCR	
VEN	275,857	4.541	178’237		54,975
BR	395,849	3.981	2,272	Dominated by VEN	
VEN + R	433,489	6.581	224,564		77,286
Ibrutinib	523,146	4.450	-78,132	Dominated by VEN and VEN+R	
Ibrutinib+BR	614,999	5.303	-84,731	Dominated by VEN+R	

Abbreviations: *B* Bendamustine, *C* Cyclophosphamide; *CHF* Swiss Francs, *F* Fludarabine, *ICER* Incremental cost-effectiveness ratio, *NMB* Net monetary benefit, *QALY* Quality-adjusted life year, *R* Rituximab, *VEN* Venetoclax
[1]Assuming a WTP threshold of CHF 100,000 / QALY
[2]ICER for undominated adjacent pair of therapies (in CHF/QALY gained)

Table S18 Scenario analysis for VEN+R versus comparators (A)

	VEN+R vs Ibrutinib			VEN+R vs Idelalisib+R			VEN+R vs FCR		
	**Inc. costs**	**Inc. QALYs**	**ICER***	**Inc. costs**	**Inc. QALYs**	**ICER***	**Inc. costs**	**Inc. QALYs**	**ICER***
**Base case**	**-185,679**	**2.130**	**Dominated by VEN+R (D)**	**75,178**	**4.101**	**18,329**	**146,219**	**3.667**	**39,879**
Discount rate. Costs: 0%, QALYs: 0%	-220,119	3.096	D	81,469	5.560	14,652	155,112	5.048	30,730
Discount rate. Costs: 0%, QALYs: 6%	-220,119	1.533	D	81,469	3.147	25,886	155,112	2.773	55,942
Discount rate. Costs: 6%, QALYs: 6%	-157,568	1.533	D	71,203	3.147	22,624	139,790	2.773	50,416
Discount rate. Costs: 6%, QALYs: 0%	-157,568	3.096	D	71,203	5.560	12,806	139,790	5.048	27,694
Time horizon: 1 year	25,106	0.004	5,605,411	40,973	0.040	1,036,365	73,194	0.027	2,720,745
Time horizon: 2 year	29,007	0.037	788,972	83,804	0.185	453,142	139,023	0.126	1,099,864
Time horizon: 5 year	-96,141	0.280	D	66,761	0.999	66,830	138,310	0.764	180,937
Time horizon: 10 year	-172,514	0.917	D	67,536	2.424	27,864	139,070	2.035	68,355
Time horizon: 15 year	-186,257	1.489	D	70,825	3.329	21,274	141,962	2.901	48,935
Time horizon: 25 year	-186,372	2.046	D	74,382	4.013	18,536	145,424	3.578	40,644
PFS/OS extrapolation: Generalised Gamma	-170,490	2.071	D	76,486	4.033	18,963	145,838	3.592	40,603
PFS/OS extrapolation: Gamma	-208,983	2.232	D	74,695	4.315	17,309	146,112	3.864	37,818
PFS/OS extrapolation: Log-logistic	-242,933	2.290	D	72,551	4.364	16,624	147,017	3.931	37,398
PFS/OS extrapolation: Log-normal	-297,379	2.480	D	73,571	4.842	15,193	145,273	4.350	33,395
Individual curve estimation for PFS and OS (adjusted)	-262,468	0.786	D	81,152	4.241	19,133	147,043	3.015	48,770
Individual curve estimation for PFS and OS (unadjusted)	-186,678	2.060	D	83,334	4.422	18,845	147,104	3.034	48,481
TLS prophylaxis cost halved	-189,900	2.130	D	70,956	4.101	17,300	141,997	3.667	38,728
TLS prophylaxis cost doubled	-177,235	2.130	D	83,621	4.101	20,388	154,662	3.667	42,182
TLS prophylaxis cost removed	-194,122	2.130	D	66,735	4.101	16,271	137,775	3.667	37,576
Pre and post-progression routine costs of care halved	-187,435	2.130	D	72,320	4.101	17,633	143,554	3.667	39,152
Pre and post-progression routine costs of care doubled	-182,166	2.130	D	80,894	4.101	19,723	151,548	3.667	41,333
Pre and post-progression routine costs of care removed	-189,191	2.130	D	69,462	4.101	16,936	140,889	3.667	38,425
Pre and post-progression routine costs of care frequency from ibrutinib submission	-185,607	2.130	D	75,490	4.101	18,405	146,459	3.667	39,944
Terminal care cost + 5%	-185,788	2.130	D	75,001	4.101	18,286	146,055	3.667	39,834
Terminal care cost + 10%	-185,897	2.130	D	74,824	4.101	18,243	145,891	3.667	39,790
Terminal care cost + 15%	-186,006	2.130	D	74,646	4.101	18,200	145,727	3.667	39,745
Terminal care cost + 20%	-185,940	2.130	D	74,753	4.101	18,226	145,825	3.667	39,772

	VEN+R vs Ibrutinib			VEN+R vs Idelalisib+R			VEN+R vs FCR		
	Inc. costs	Inc. QALYs	ICER*	Inc. costs	Inc. QALYs	ICER*	Inc. costs	Inc. QALYs	ICER*
**Base case**	**-185,679**	**2.130**	**Dominated by VEN+R (D)**	**75,178**	**4.101**	**18,329**	**146,219**	**3.667**	**39,879**
VEN+R and BR follow observed ToT from MURANO	-190,659	2.130	D	70,198	4.101	17,115	141,238	3.667	38,521
All treatments use rapid IV infusion of Rituximab	-186,056	2.130	D	75,159	4.101	18,325	146,181	3.667	39,869
Adverse event rates halved	-185,834	2.132	D	74,960	4.103	18,270	146,129	3.667	39,853
Adverse event rates doubled	-185,367	2.127	D	75,614	4.099	18,449	146,398	3.666	39,931
Adverse events removed	-185,990	2.134	D	74,742	4.104	18,210	146,039	3.667	39,827
Utilities: Dretzke et al (PFS:0.800, PPS:0.600)	-185,679	2.167	D	75,178	4.291	17,521	146,219	3.809	38,387
Utilities: Beusterien et al (PFS:0.819, PPS:0.680)	-185,679	2.395	D	75,178	4.544	16,544	146,219	4.077	35,867
Diff. between pre and post-progression utility: 0.1	-185,679	2.259	D	75,178	4.212	17,848	146,219	3.796	38,520
Diff. between pre and post-progression utility: 0.2	-185,679	1.991	D	75,178	3.982	18,881	146,219	3.526	41,464
Diff. between pre and post-progression utility: 0.3	-185,679	1.722	D	75,178	3.751	20,040	146,219	3.257	44,894
Diff. between pre and post-progression utility: 0.4	-185,679	1.453	D	75,178	3.521	21,352	146,219	2.987	48,944
Diff. between pre and post-progression utility: 0.5	-185,679	1.184	D	75,178	3.291	22,847	146,219	2.718	53,797
Disutilities doubled	-185,679	2.127	D	75,178	4.099	18,342	146,219	3.666	39,882
Disutilities removed	-185,679	2.134	D	75,178	4.104	18,317	146,219	3.667	39,876

*VEN+R vs comparator

Abbreviations: *B* Bendamustine, *D* Dominated by VEN+R, *Diff* Difference, *ICER* Incremental cost-effectiveness ratio, *Inc* Incremental, *OS* Overall survival, *QALY* Quality adjusted life year, *PFS* Progression-free survival, *PPS* Post-progression survival, *R* Rituximab, *VEN* Venetoclax

Table S19 Scenario analysis for VEN+R versus comparators (B)

	VEN+R vs BR			VEN+R vs VEN monotherapy			VEN+R vs Ibrutinib+BR		
	**Inc. costs**	**Inc. QALYs**	**ICER***	**Inc. costs**	**Inc. QALYs**	**ICER***	**Inc. costs**	**Inc. QALYs**	**ICER***
**Base case**	**147,851**	**2.599**	**56,881**	**-86,001**	**2.040**	**Dominated by VEN+R (D)**	**-314,390**	**1.278**	**Dominated by VEN+R (D)**
Discount rate. Costs: 0%, QALYs: 0%	155,117	3.648	42,524	-98,047	2.932	D	-380,871	1.925	D
Discount rate. Costs: 0%, QALYs: 6%	155,117	1.938	80,051	-98,047	1.486	D	-380,871	0.890	D
Discount rate. Costs: 6%, QALYs: 6%	142,416	1.938	73,496	-75,063	1.486	D	-263,972	0.890	D
Discount rate. Costs: 6%, QALYs: 0%	142,416	3.648	39,042	-75,063	2.932	D	-263,972	1.925	D
Time horizon: 1 year	76,588	0.024	3,138,195	18,671	0.010	1,788,050	-74	0.004	D
Time horizon: 2 year	143,268	0.106	1,353,352	28,202	0.051	553,658	676	0.017	38,799
Time horizon: 5 year	143,836	0.541	265,959	-59,448	0.325	D	-146,354	0.125	D
Time horizon: 10 year	143,326	1.336	107,314	-87,849	0.943	D	-265,963	0.454	D
Time horizon: 15 year	144,617	1.947	74,291	-88,665	1.455	D	-303,448	0.802	D
Time horizon: 25 year	147,118	2.516	58,472	-86,666	1.960	D	-314,466	1.206	D
PFS/OS extrapolation: Generalised Gamma	147,350	2.566	57,426	-80,610	1.967	D	-285,301	1.254	D
PFS/OS extrapolation: Gamma	147,796	2.824	52,334	-94,553	2.152	D	-355,003	1.315	D
PFS/OS extrapolation: Log-logistic	148,831	2.815	52,870	-104,553	2.252	D	-399,668	1.354	D
PFS/OS extrapolation: Log-normal	147,026	3.158	46,555	-133,167	2.454	D	-450,184	1.482	D
Individual curve estimation for PFS and OS (adjusted)	147,809	2.715	54,442	-44,091	2.954	D	-292,137	0.834	D
Individual curve estimation for PFS and OS (unadjusted)	147,809	2.715	54,442	-43,855	3.244	D	-215,564	1.193	D
TLS prophylaxis cost halved	143,630	2.599	55,256	-86,007	2.040	D	-318,611	1.278	D
TLS prophylaxis cost doubled	156,294	2.599	60,129	-85,990	2.040	D	-305,947	1.278	D
TLS prophylaxis cost removed	139,408	2.599	53,632	-86,013	2.040	D	-322,833	1.278	D
Pre and post-progression routine costs of care halved	146,258	2.599	56,268	-87,381	2.040	D	-315,658	1.278	D
Pre and post-progression routine costs of care doubled	151,038	2.599	58,106	-83,242	2.040	D	-311,853	1.278	D
Pre and post-progression routine costs of care removed	144,665	2.599	55,655	-88,761	2.040	D	-316,927	1.278	D
Pre and post-progression routine costs of care frequency from ibrutinib submission	148,144	2.599	56,993	-85,818	2.040	D	-314,427	1.278	D
Terminal care cost + 5%	147,741	2.599	56,838	-86,096	2.040	D	-314,467	1.278	D
Terminal care cost + 10%	147,630	2.599	56,795	-86,190	2.040	D	-314,545	1.278	D
Terminal care cost + 15%	147,519	2.599	56,753	-86,285	2.040	D	-314,622	1.278	D
Terminal care cost + 20%	147,586	2.599	56,778	-86,228	2.040	D	-314,576	1.278	D

	VEN+R vs BR			VEN+R vs VEN monotherapy			VEN+R vs Ibrutinib+BR		
	**Inc. costs**	**Inc. QALYs**	**ICER***	**Inc. costs**	**Inc. QALYs**	**ICER***	**Inc. costs**	**Inc. QALYs**	**ICER***
**Base case**	**147,851**	**2.599**	**56,881**	**-86,001**	**2.040**	**Dominated by VEN+R (D)**	**-314,390**	**1.278**	**Dominated by VEN+R (D)**
VEN+R and BR follow observed ToT from MURANO	144,041	2.599	55,415	-90,982	2.040	D	-319,370	1.278	D
All treatments use rapid IV infusion of Rituximab	147,807	2.599	56,864	-86,379	2.040	D	-314,389	1.278	D
Adverse event rates halved	148,061	2.599	56,962	-85,939	2.040	D	-314,199	1.277	D
Adverse event rates doubled	147,431	2.599	56,717	-86,127	2.039	D	-314,772	1.279	D
Adverse events removed	148,271	2.599	57,044	-85,876	2.040	D	-314,007	1.277	D
Utilities: Dretzke et al (PFS:0.800, PPS:0.600)	147,851	2.783	53,126	-86,001	2.152	D	-314,390	1.247	D
Utilities: Beusterien et al (PFS:0.819, PPS:0.680)	147,851	2.844	51,980	-86,001	2.249	D	-314,390	1.465	D
Diff. between pre and post-progression utility: 0.1	147,851	2.596	56,956	-86,001	2.073	D	-314,390	1.416	D
Diff. between pre and post-progression utility: 0.2	147,851	2.603	56,799	-86,001	2.003	D	-314,390	1.128	D
Diff. between pre and post-progression utility: 0.3	147,851	2.610	56,643	-86,001	1.933	D	-314,390	0.840	D
Diff. between pre and post-progression utility: 0.4	147,851	2.617	56,488	-86,001	1.862	D	-314,390	0.553	D
Diff. between pre and post-progression utility: 0.5	147,851	2.625	56,333	-86,001	1.792	D	-314,390	0.265	D
Disutilities doubled	147,851	2.599	56,879	-86,001	2.039	D	-314,390	1.279	D
Disutilities removed	147,851	2.599	56,883	-86,001	2.040	D	-314,390	1.277	D

*VEN+R vs comparator

Abbreviations: *B* Bendamustine, *D* Dominated by VEN+R, *Diff* Difference, *ICER* Incremental cost-effectiveness ratio, *Inc* Incremental, *OS* Overall survival, *QALY* Quality adjusted life year, *PFS* Progression-free survival, *PPS* Post-progression survival, *R* Rituximab, *VEN* Venetoclax

Table S20 Per patient budget impact by cost category over 5 years

Cost category	World with VEN+R (in CHF)	World without VEN+R (in CHF)	Budget impact (in CHF)
**Total costs**	**219,018**	**224,929**	**-5,912**
Active treatment	202,751	210,588	-7,837
Treatment admin	1,740	1,401	339
Wastage	886	641	245
PFS health state costs	3,098	2,658	440
PPS health state costs	714	911	-197
Terminal care costs	5,522	7,741	-2,219
Treatment specific monitoring (e.g. TLS)	3,805	611	3,195
Adverse events	501	378	123

Abbreviations: CHF, Swiss Francs; PFS, Progression-free survival ; OS, Overall survival; R, Rituximab; VEN, Venetoclax

Table S21 Budget impact scenario analysis results

	Absolute budget impact						Percent budget impact (% change from base case)					
	**Year 1 (CHF)**	**Year 2 (CHF)**	**Year 3 (CHF)**	**Year 4 (CHF)**	**Year 5 (CHF)**	**Total (CHF)**	**Year 1**	**Year 2**	**Year 3**	**Year 4**	**Year 5**	**Total**
**Base case**	**9,572,338**	**10,881,413**	**-11,463,419**	**-11,050,542**	**-10,249,259**	**-12,309,469**						
Venetoclax price: +5%	10,597,504	12,209,035	-11,255,662	-10,831,133	-9,974,015	**-9,254,271**	10.71%	12.20%	-1.81%	-1.99%	-2.69%	25%
Venetoclax price: +10%	11,622,670	13,536,660	-11,047,900	-10,611,716	-9,698,762	**-6,199,048**	21.42%	24.40%	-3.62%	-3.97%	-5.37%	50%
Venetoclax price: +15%	12,647,837	14,864,284	-10,840,139	-10,392,299	-9,423,509	**-3,143,826**	32.13%	36.60%	-5.44%	-5.96%	-8.06%	74%
Venetoclax price: +20%	13,673,003	16,191,908	-10,632,377	-10,172,883	-9,148,256	**-88,604**	42.84%	48.80%	-7.25%	-7.94%	-10.74%	99%
Venetoclax price: -5%	8,547,171	9,553,786	-11,671,186	-11,269,966	-10,524,521	**-15,364,715**	-10.71%	-12.20%	1.81%	1.99%	2.69%	-25%
Venetoclax price: -10%	7,522,005	8,226,162	-11,878,947	-11,489,382	-10,799,774	**-18,419,937**	-21.42%	-24.40%	3.62%	3.97%	5.37%	-50%
Venetoclax price: -15%	6,496,838	6,898,538	-12,086,709	-11,708,799	-11,075,027	**-21,475,160**	-32.13%	-36.60%	5.44%	5.96%	8.06%	-74%
Venetoclax price: -20%	5,471,672	5,570,913	-12,294,471	-11,928,216	-11,350,281	**-24,530,382**	-42.84%	-48.80%	7.25%	7.94%	10.74%	-99%
Routine costs of care: +5%	9,572,574	10,882,584	-11,460,654	-11,045,505	-10,241,872	**-12,292,873**	0.00%	0.01%	-0.02%	-0.05%	-0.07%	0%
Routine costs of care: +10%	9,572,811	10,883,755	-11,457,889	-11,040,468	-10,234,484	**-12,276,277**	0.00%	0.02%	-0.05%	-0.09%	-0.14%	0%
Routine costs of care: +15%	9,573,047	10,884,925	-11,455,125	-11,035,431	-10,227,097	**-12,259,681**	0.01%	0.03%	-0.07%	-0.14%	-0.22%	0%
Routine costs of care: +20%	9,573,284	10,886,096	-11,452,360	-11,030,395	-10,219,710	**-12,243,085**	0.01%	0.04%	-0.10%	-0.18%	-0.29%	1%
Routine costs of care: -5%	9,572,101	10,880,242	-11,466,183	-11,055,578	-10,256,646	**-12,326,065**	0.00%	-0.01%	0.02%	0.05%	0.07%	0%
Routine costs of care: -10%	9,571,864	10,879,071	-11,468,948	-11,060,615	-10,264,034	**-12,342,661**	0.00%	-0.02%	0.05%	0.09%	0.14%	0%
Routine costs of care: -15%	9,571,628	10,877,901	-11,471,713	-11,065,652	-10,271,421	**-12,359,257**	-0.01%	-0.03%	0.07%	0.14%	0.22%	0%
Routine costs of care: -20%	9,571,391	10,876,730	-11,474,477	-11,070,689	-10,278,808	**-12,375,853**	-0.01%	-0.04%	0.10%	0.18%	0.29%	-1%
Adverse event rates halved	9,521,841	10,876,956	-11,467,901	-11,055,311	-10,254,221	**-12,378,637**	-0.53%	-0.04%	0.04%	0.04%	0.05%	-1%
Adverse event rates doubled	9,673,332	10,890,328	-11,454,454	-11,041,003	-10,239,334	**-12,171,132**	1.06%	0.08%	-0.08%	-0.09%	-0.10%	1%
Adverse events removed	9,471,344	10,872,498	-11,472,384	-11,060,081	-10,259,184	**-12,447,806**	-1.06%	-0.08%	0.08%	0.09%	0.10%	-1%
Cost of TLS prophylaxis: -25%	8,994,574	10,816,863	-11,543,084	-11,144,573	-10,353,837	**-13,230,058**	-6.04%	-0.59%	0.69%	0.85%	1.02%	-7%
Cost of TLS prophylaxis: -50%	8,416,810	10,752,313	-11,622,749	-11,238,605	-10,458,415	**-14,150,646**	-12.07%	-1.19%	1.39%	1.70%	2.04%	-15%
Cost of TLS prophylaxis:+25%	10,150,101	10,945,963	-11,383,754	-10,956,510	-10,144,681	**-11,388,880**	6.04%	0.59%	-0.69%	-0.85%	-1.02%	7%
Cost of TLS prophylaxis:+50%	10,727,865	11,010,513	-11,304,088	-10,862,479	-10,040,103	**-10,468,291**	12.07%	1.19%	-1.39%	-1.70%	-2.04%	15%
Treated R/R population: +10%	10,529,571	11,969,554	-12,609,761	-12,155,596	-11,274,185	**-13,540,416**	10.00%	10.00%	10.00%	10.00%	10.00%	-10%
Treated R/R population:-10%	8,615,104	9,793,272	-10,317,077	-9,945,488	-9,224,333	**-11,078,522**	-10.00%	-10.00%	-10.00%	-10.00%	-10.00%	10%
Include only prevalent patients	8,711,013	8,801,359	-12,965,661	-11,885,972	-10,141,382	**-17,480,643**	-9.00%	-19.12%	13.10%	7.56%	-1.05%	-42%
Include only incident patients	861,325	2,080,054	1,502,242	835,430	-107,877	**5,171,174**	-91.00%	-80.88%	-113.1%	-107.6%	-98.95%	142%

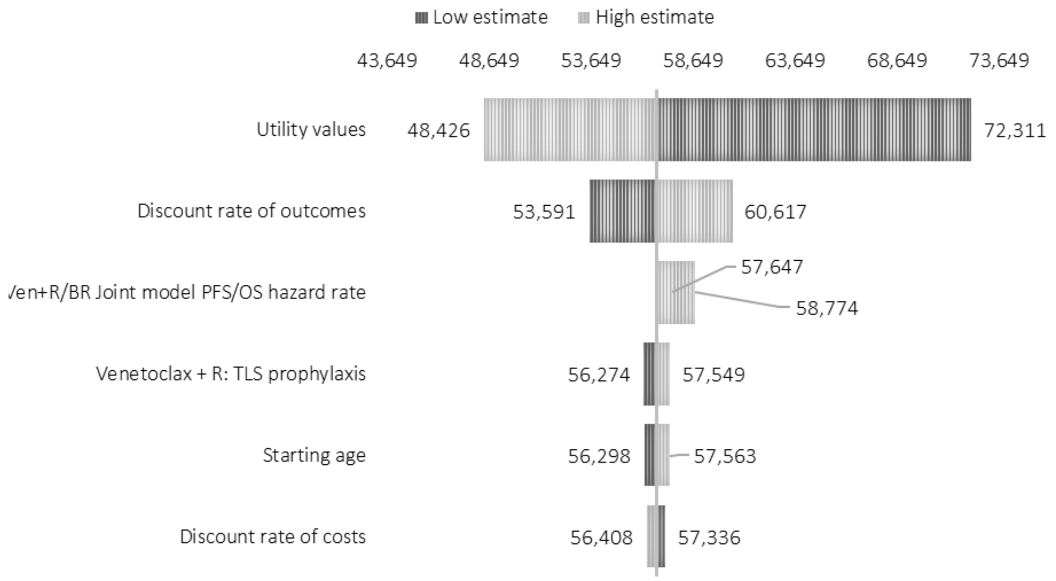

Abbreviations: *CHF* Swiss Francs, *ICER* Incremental cost-effectiveness ratio, *OS* Overall Survival, *PFS* Progression-free survival, *R* Rituximab, *TLS* Tumour Lysis Syndrome, *VEN* Venetoclax

Figure S1 Tornado plot of parameter impact on incremental ICER (in CHF/QALY, VEN+R vs BR)

References

1. Seymour, J.F., et al., *Venetoclax–Rituximab in Relapsed or Refractory Chronic Lymphocytic Leukemia.* New England Journal of Medicine, 2018. **378**(12): p. 1107-1120.
2. ClinicalTrials.gov, *A phase 2 open-label study of the efficacy and safety of ABT-199 (GDC-0199) in chronic lymphocytic leukemia (CLL) subjects with relapse or refractory to B-cell receptor signalling pathway inhibitor therapy*. 2016.
3. Stilgenbauer, S., et al., *Venetoclax in relapsed or refractory chronic lymphocytic leukaemia with 17p deletion: a multicentre, open-label, phase 2 study.* The lancet oncology, 2016. **17**(6): p. 768-778.
4. Roberts, A.W., et al., *Targeting BCL2 with venetoclax in relapsed chronic lymphocytic leukemia.* New England Journal of Medicine, 2016. **374**(4): p. 311-322.
5. Byrd, J.C., et al., *Long-term efficacy and safety with ibrutinib (ibr) in previously treated chronic lymphocytic leukemia (CLL): Up to four years follow-up of the RESONATE study.* Journal of Clinical Oncology, 2017. **35**(15_suppl): p. 7510-7510.
6. Byrd, J.C., et al., *Ibrutinib versus Ofatumumab in Previously Treated Chronic Lymphoid Leukemia.* New England Journal of Medicine, 2014. **371**(3): p. 213-223.
7. Badoux, X.C., et al., *Fludarabine, cyclophosphamide, and rituximab chemoimmunotherapy is highly effective treatment for relapsed patients with CLL.* Blood, The Journal of the American Society of Hematology, 2011. **117**(11): p. 3016-3024.
8. Chanan-Khan, A., et al., *Ibrutinib combined with bendamustine and rituximab compared with placebo, bendamustine, and rituximab for previously treated chronic lymphocytic leukaemia or small lymphocytic lymphoma (HELIOS): a randomised, double-blind, phase 3 study.* The Lancet Oncology, 2016. **17**(2): p. 200-211.
9. Sharman, J.P., et al., *Second Interim Analysis of a Phase 3 Study of Idelalisib (ZYDELIG®) Plus Rituximab (R) for Relapsed Chronic Lymphocytic Leukemia (CLL): Efficacy Analysis in Patient Subpopulations with Del(17p) and Other Adverse Prognostic Factors.* Blood, 2014. **124**(21): p. 330-330.
10. Furman, R.R., et al., *Idelalisib and rituximab in relapsed chronic lymphocytic leukemia.* New England Journal of Medicine, 2014. **370**(11): p. 997-1007.

11. National Institute for Health and Care Excellence, *Single Technology Appraisal (TA561). Venetoclax in combination with rituximab for treating relapsed or refractory chronic lymphocytic leukaemia [ID 1097]*. 2018.
12. National Institute for Health and Care Excellence, *TA359: Idelalisib for treating chronic lymphocytic leukaemia*. 2015.
13. Dretzke, J., et al., *Rituximab for the treatment of relapsed/refractory chronic lymphocytic leukaemia.* Health Technol Assess, 2010. **14**(Suppl. 2): p. 19-26.
14. National Institute for Health and Care Excellence, *TA487: Venetoclax for treating chronic lymphocytic leukaemia [ID944]*. 2017.
15. Swiss Federal Office. *Spezialitätenliste*. 5.8.2020]; Available from: http://www.spezialitaetenliste.ch/ShowPreparations.aspx.
16. Deutsche Gesellschaft für Hämatologie und Medizinische Onkologie e.V. *Onkopedia Leitlinie Chronische Lymphatische Leukämie (CLL)*. 17.8.2021]; Available from: https://www.onkopedia.com/de/onkopedia/guidelines/chronische-lymphatische-leukaemie-cll/@@guideline/html/index.html.
17. Swiss Federal Statistical Office. *Population growth in 2019: provisional results: Switzerland's population continued to increase and age in 2019*. 10.9.2020]; Available from: https://www.bfs.admin.ch/bfs/en/home/statistics/population.assetdetail.12247184.html.
18. Swiss Federal Statistical Office. *Population change in Switzerland in 2019: definitive figures: Stable demographic growth of 0.7% in 2019*. 10.9.2020]; Available from: https://www.bfs.admin.ch/bfs/en/home/statistics/population.assetdetail.13667088.html.
19. Zoellner, A.K., et al., *Altered treatment of chronic lymphocytic leukemia in Germany during the last decade.* Ann Hematol, 2016. **95**(6): p. 853-61.
20. IQWiG, *IQWiG assessment report. Venetoclax (chonische lymphatische Leukämie). Bericht Nummer 498. Auftrag G16-14*. 2017.
